# Collateral effects of COVID-19 stay-at-home orders on violence against women in the United States, January 2019 to December 2020

**DOI:** 10.1186/s12889-023-17546-y

**Published:** 2024-01-02

**Authors:** Patricia C. Lewis, Yuk Fai Cheong, Nadine J. Kaslow, Kathryn M. Yount

**Affiliations:** 1 Department of Health Sciences, Sacred Heart University, Fairfield, 06825 USA; 2 Department of Psychology, Emory University, Atlanta, 30322 USA; 3 Department of Psychiatry and Behavioral Sciences, Emory University, Atlanta, 30322 USA; 4 Hubert Department of Global Health and Department of Sociology, Emory University, Atlanta, 30322 USA

**Keywords:** Violence against women, COVID-19, Femicide, Intimate partner violence, Non-pharmaceutical risk mitigation strategies, United States

## Abstract

**Background:**

The necessary execution of non-pharmaceutical risk-mitigation (NPRM) strategies to reduce the transmission of COVID-19 has created an unprecedented natural experiment to ascertain whether pandemic-induced social-policy interventions may elevate collateral health risks. Here, we assess the effects on violence against women (VAW) of the duration of NPRM measures that were executed through jurisdictional-level orders in the United States. We expect that stay-at-home orders, by reducing mobility and disrupting non-coresident social ties, are associated with higher incident reporting of VAW.

**Methods:**

We used aggregate data from the Murder Accountability Project from January 2019 through December 2020, to estimate count models examining the effects of the duration of jurisdictional-level (*N* = 51) stay-at-home orders on femicide. Additionally, we used data from the National Incident-Based Reporting System to estimate a series of count models that examined the effects of the duration of jurisdictional-level (*N* = 26) stay-at-home orders on non-lethal violence against women, including five separate measures of intimate partner violence (IPV) and a measure of non-partner sexual violence.

**Results:**

Results from the count models indicated that femicide was not associated with COVID-19 mitigation strategies when adjusted for seasonal effects. However, we found certain measures of non-lethal VAW to be significantly associated in adjusted models. Specifically, reported physical and economic IPV were positively associated with stay-at-home orders while psychological IPV and non-partner sexual violence were negatively associated with stay-at-home orders. The combination measure of all forms of IPV was positively associated with the duration of stay-at-home orders, indicating a net increase in risk of IPV during lockdowns.

**Conclusions:**

The benefits of risk-mitigation strategies to reduce the health impacts directly associated with a pandemic should be weighed against their costs with respect to women’s heightened exposure to certain forms of violence and the potentially cascading impacts of such exposure on health. The effects of COVID-19 NPRM strategies on IPV risk nationally and its immediate and long-term health sequelae should be studied, with stressors like ongoing pandemic-related economic hardship and substance misuse still unfolding. Findings should inform the development of social policies to mitigate the collateral impacts of crisis-response efforts on the risk of VAW and its cascading sequelae.

**Supplementary Information:**

The online version contains supplementary material available at 10.1186/s12889-023-17546-y.

## Background

Before the COVID-19 pandemic, violence against women (VAW) in the United States (U.S.) was understood to be a pervasive public health problem, with one in three women reporting intimate partner violence (IPV) and about half of all women reporting some form of sexual violence in their lifetime [1]. Moreover, crime data reported to the Federal Bureau of Investigation (FBI) indicated that 1,795 women were murdered by men (femicide) in 2019 alone, and most of these victims were known to the perpetrator [2]. When the World Health Organization pronounced COVID-19 to be a global pandemic in March of 2020, governments around the world began to enact emergency policies, including social distancing measures and more extreme “lockdowns” (e.g. stay-at-home orders) to prevent viral spread and associated morbidity and mortality. A concurrent public-health concern was that COVID-19 risk-mitigation strategies had the potential to increase the risk of VAW [3–5]. In a public video message, the United Nations Secretary Guterres’ asserted: “We know lockdowns and quarantines are essential to suppressing COVID-19, but they can trap women with abusive partners." In the U.S., very early media reports emphasized a rise in domestic violence hotline calls^1^ [6, 7]. Since then, evidence has accumulated of elevated levels of VAW, particularly IPV, in communities across the U.S. during the early months of the pandemic [8–11]. This violence coupled with the disparate economic impacts of the COVID-19 pandemic on women have the potential to reverse the limited progress made on curbing VAW during recent decades [12].

Risk factors for VAW are rooted in broader structural and social inequities [13–17]. As such, the risk of such violence often is heightened in complex emergency situations that disrupt social processes and systems, as in the case of conflicts, pandemics, or other humanitarian crises [5, 18]. During the 2014–2016 Ebola epidemic in West Africa, for example, the United Nations Development Programme [19] noted increased reporting of gender-based violence (term used by the authors), which they attributed to increased economic strain and quarantine measures. In the COVID-19 pandemic specifically, the combination of stay-at-home orders and the economic impact of the crisis could exacerbate factors associated with risk of VAW which, during normal circumstances, include men’s unemployment, housing insecurity, low wages, childcare strains, social isolation, and other predisposing personal factors such as histories of child maltreatment against men [20, 21]. The economic strain apparent in the early pandemic [22] also may have adversely affected situational coping mechanisms, such as anxiety, depression, and substance abuse, which heighten the immediate risk for IPV perpetration [23–25].

A major concern of pandemic risk-mitigation strategies is their intensification of the social isolation that victims of violence typically experience. Social isolation is a known risk factor for VAW and often is used as a tactic of abuse and control in intimate partnerships [26]. Social isolation, in the case of social distancing measures, may not be of the perpetrator's design but can operate through multiple pathways to influence the same outcomes [5, 27]. Isolation separates people from resources and social support networks in the community, and such isolation heightens the risk of VAW and limits the capacity of victims to seek help. Stay-at-home orders also increase the time spent with potential perpetrators, and studies on IPV during times of crisis, primarily among refugees, suggest that increased time spent at home with family under stress increases the risk of IPV [28–30].

During the calendar year in which COVID-19 mitigation strategies were implemented (2020), the rate of documented femicides [31] in the U.S. increased by 14%, from 1.18 per 100,000 females in 2019 to 1.34 per 100,000 females in 2020 [31]. A recent meta-analysis of VAW during the COVID-19 pandemic also suggests an increase in reported incidents of IPV in the U.S. [11]. While a few studies indicate no increase in IPV [32, 33] or even a decrease [34], Piquero and colleagues’ [11] review indicated an overall increase in IPV, with a mean effect size of 0.87 in the U.S. Importantly, prior studies looking at the impact of COVID-19 on VAW in the U.S. had been limited in scope to a single city with multiple measures of violence or several cities but with only one measure of IPV [8, 35, 36], which may have misrepresented the actual nature of the pandemic’s effects on violence against women [37].

Only recently have population-level data for the U.S. become available on rates of femicide and reported non-lethal VAW. To advance our understanding of the effects of the COVID-19 pandemic on VAW, the present analysis draws from community or city-level data to assess the effects on reported incidents of VAW of the timing and duration of non-pharmaceutical risk mitigation (NPRM) measures that were executed through state-level orders in the U.S. NRPM, also known as community mitigation strategies, refer to actions apart from getting vaccinated or taking medication, that individuals and communities can embrace to slow the spread of a virus, such as SARS-CoV2. While these strategies show efficacy in controlling a pandemic [38], some of these interventions have the potential to cause collateral harm [39]. One such community mitigation strategy employed in the U.S. was stay-at-home orders, which were effective at reducing population movement [40] and close person-to-person contact outside the home [41], and thus, decreased community spread of the virus [42]. We expect that the same stay-at-home orders - by reducing mobility and disrupting non-coresident social ties-were associated with a higher incidence of VAW in women’s homes. However, despite the expected increase in non-lethal and lethal forms of VAW during this acute period of crisis, the social isolation and breakdown of municipal operations may have set the stage for a decrease in reporting non-lethal forms of VAW. Furthermore, with limited resources for non-COVID-related autopsies during the early pandemic [43, 44], femicides might not have been accurately recorded. Therefore, findings from this study can add to a growing body of literature on likely lower bounds of the impact of COVID-NPRM strategies on rates of violence against women. Conclusions from this study can be used to guide policies about the supports that are needed during pandemics or other crises that mitigate the risks of VAW and provide the necessary resources for individuals who currently are impacted by VAW.

## Methods

In this secondary data analysis, we assembled quarterly (three-monthly) homicide data from the Murder Accountability Project (MAP) [45] from January 2019 through December 2020 to estimate count models examining the effects of the duration in days of jurisdictional-level (*N* = 51) stay-at-home orders on female homicide. Crime data from the MAP is the most comprehensive data on homicides in the U.S., as it includes homicides reported to the FBI as well as data obtained through the Freedom of Information Act from jurisdictions that do not report homicides to the Department of Justice. Because homicides by unknown offenders may be misclassified and low counts of femicide preclude disaggregation, we defined femicide as including all homicides of female victims, regardless of the age of the victim or relationship to the perpetrator.

We also utilized data from the National Incident-Based Reporting System (NIBRS) [46] to estimate a series of count models that examine the effects of the duration in days of jurisdictional-level stay-at-home orders on non-lethal VAW across the same calendar period. The NIBRS’s victim segment data include information on the victim’s age, sex as defined at birth, race, and relationship to the perpetrator(s), making it an ideal dataset to study national incidences of reported VAW, particularly IPV. We included five measures of non-lethal IPV committed by a current or former intimate partner: physical violence (including aggravated assault or simple assault); psychological violence (including any crime that involved intimidation); economic violence (including larceny, robbery, destruction of property, fraud, etc.); sexual violence (including fondling, commercial sex acts, incest, rape, sexual assault with an object, sodomy, or statutory rape); and a combined measure of these four forms of IPV. We also included a measure of sexual violence committed by a non-partner, inclusive of incidents in which the offender was unknown. Our approach to include various forms of non-lethal IPV in this study is grounded in the understanding that IPV is a multifaceted phenomenon [37, 47, 48], with each form presenting unique challenges and consequences. The implementation of COVID-19 mitigation strategies, such as stay-at-home orders, likely had differential impacts on these various forms of IPV. By incorporating multiple measures of IPV - physical, emotional, sexual, economic, and psychological - we aimed to capture a comprehensive, holistic, and nuanced view of the IPV experiences during the pandemic. To accommodate the potential for overlap between different forms of IPV, we also assessed a combined measure of any IPV. This approach allows us to examine both specific and overall patterns of IPV, providing a more complete understanding of its dynamics under the unique conditions imposed by COVID-19 mitigation strategies.

Whereas MAP data on female homicides were available for all 50 states and the District of Columbia, the NIBRS data is incomplete in that not every state reports crime to the NIBRS system. For instance, seven states - Alaska, California, Florida, Nevada, New Jersey, New York, and Pennsylvania - did not report crimes to NIBRS for the entire 24-month period in question. In addition, of those states that did report crimes to NIBRS, some states had very few agencies or regions reporting. For the main analysis, we included only those states that reported data to the NIBRS all 24 months with at least 70% of the state’s population being included in the catchment area of agencies reporting (*N* = 26 states or jurisdictions). Details on state inclusion criteria are available in Appendix A. Supplemental sensitivity analyses included jurisdictions in which 80% of the population was included in the catchment area and, separately, 60% of the population. In the first sensitivity analyses (Table B1 in the Appendix), the criterion for jurisdiction inclusion was lowered to 60% of the population being included in the catchment area, which yielded a sample size of 28 jurisdictions. In the second sensitivity analysis (Table B2), the inclusion criterion was set at 80% of the population being included in the catchment area and yielded a sample size of 22 jurisdictions.

Finally, policy data from BallotPedia [49] captured the quarterly duration in days of jurisdiction-level stay-at-home executive orders put in place in response to the COVID-19 pandemic in 2020. Those jurisdictions that did not enact a stay-at-home executive order were considered to have zero (0) days of duration. All other jurisdictions had quarterly exposure measures of 1-90 days of stay-at-home order implementation.

Outcomes of interest were jurisdiction-specific quarterly counts of femicide, and separately, of non-lethal VAW (5 measures of IPV and 1 measure of non-partner sexual violence). In descriptive analyses, quarterly counts of femicide and non-lethal IPV were averaged for jurisdictions, stratified by the total number of days in 2020 in which a jurisdictional stay-at-home order was in place (no order, 1-45 days, 46-60 days, > 60 days). The number of jurisdictions within each stratum varied between 8 and 17 within the femicide analysis and between 5 and 7 in the non-lethal VAW analysis. The number of jurisdictions is noted in the legend of each figure. Then, to examine the effects on each outcome of the duration of jurisdictional stay-at-home order, count models were estimated, adjusting sequentially for yearly estimated jurisdictional-level female population^2^ and then for seasonality using a variable capturing three-month periods from January 1, 2019, to December 31, 2020, entered as a continuous variable in the model. Stata Version 14.2 was used for all analyses. The study was not subject to IRB approval, as fully de-identified, public-access data were used.

## Results

### Jurisdictional stay-at-home orders and femicide

**Fig. 1:**
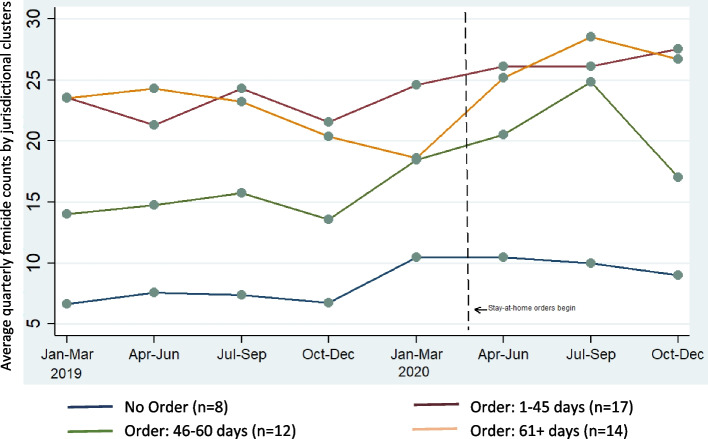
Femicide counts across jurisdictions (*N* = 51) grouped by duration of jurisdiction-level-stay-at-home executive orders, 2019–2020

The average quarterly femicide count varied across jurisdictions, with those jurisdictions that had stay-at-home orders in place showing an increase in femicide after stay-at-home orders began (March 27, 2020) (Fig. 1). For all jurisdictions, a similar seasonal pattern was apparent in 2019 and 2020; femicide counts appeared highest during July-September in both years. In the fixed-effects model unadjusted for quarter and jurisdictional population size (Table 1), the quarterly duration in days of jurisdictional stay-at-home order was positively associated with quarterly jurisdiction-specific counts of female homicide (incident rate ratio [IRR], 1.001; 95% confidence intervals [CI] 1.000, 1.003). This relationship remained the same after accounting for jurisdictional population size (IRR, 1.001; 95% CI 1.000, 1.003). However, adjusting for calendar quarter attenuated this effect (IRR, 1.000; 95% CI 0.999, 1.046).

**Table 1:** Incident rate ratios with fixed effects for quarterly jurisdiction-specific femicide counts by quarterly duration in days of jurisdiction-level-stay-at-home executive orders, 2019–2020 (*N* = 51)

	Unadjusted	Adjusted for population	Adjusted for population and seasonal effects
***Femicide***			
Stay-at-home order duration	1.001*	1.001*	1.000
Calendar Quarter			1.036***

^*^Indicates significance at the 0.05 level

^***^Indicates significance at the 0.001 level

### Jurisdictional stay-at-home orders and non-lethal reported incidents of IPV

**Fig. 2:**
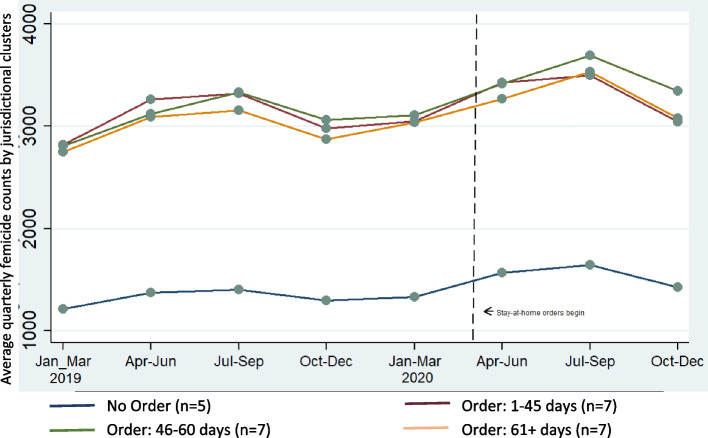
IPV counts across jurisdictions (*N* = 26) grouped by duration of jurisdiction-level-stay-at-home executive orders, 2019-2020

The average quarterly count of non-lethal reported incidents of IPV varied across jurisdictions, with all jurisdictions showing an increased incidence of IPV after COVID-19 jurisdictional-level stay-at-home orders began (March 27, 2020) (Fig. 2). For all jurisdictions, a similar seasonal pattern was apparent in 2019 and 2020; similar to seasonal patterns of femicide, counts of IPV appeared highest during July–September in both years. While those jurisdictions that did not have stay-at-home orders in place (*n* = 5) had lower counts of reported IPV, this lower count is due primarily to lower population sizes in these states than in those states that enacted stay-at-home measures.

In the fixed-effects models unadjusted for quarter and jurisdictional population size (Table 2), duration of stay-at-home order was positively associated with jurisdiction-specific counts of physical IPV (IRR, 1.001; 95% CI 1.001, 1.001), economic IPV (IRR, 1.003; 95% CI 1.003, 1.004), and the combination measure of any IPV (IRR, 1.001; 95% CI 1.001,1.002). Duration of stay-at-home orders was negatively associated with jurisdiction-specific counts of psychological IPV (IRR, 0.999; 95% CI 0.999, 0.999), sexual IPV (IRR, 0.999; 95% CI 0.998, 0.999), and non-partner sexual violence (IRR, 0.997; 95% CI 0.996, 0.997). As results in Table 2 show, the direction and significance of these relationships remained after accounting for jurisdictional variation in population size and temporal variation in calendar quarter, except in the case of sexual IPV, where the effect of stay-at-home orders was attenuated when adjusting for seasonal effects (IRR 0.999; 95% CI 0.998, 1.000).

**Table 2:** Incident rate ratios with fixed effects for quarterly jurisdiction-specific counts of non-lethal VAW by quarterly duration in days of jurisdiction-level-stay-at-home executive orders, 2019–2020 (*N* = 26)

	Unadjusted	Adjusted for population	Adjusted for population and seasonal effects
***Physical IPV***			
Stay-at-home order duration	1.001***	1.001***	1.001***
Calendar Quarter			1.013***
***Psychological IPV***			
Stay-at-home order duration	0.999***	0.999***	0.999***
Calendar Quarter			1.008***
***Economic IPV***			
Stay-at-home order duration	1.003***	1.003***	1.001***
Calendar Quarter			1.072***
***Sexual IPV***			
Stay-at-home order duration	0.999*	0.999*	0.999
Calendar Quarter			0.990*
***Any IPV***			
Stay-at-home order duration	1.001***	1.001***	1.001***
Calendar Quarter			1.018***
***Sexual Violence (Non-partner)***			
Stay-at-home order duration	0.997***	0.997***	0.997***
Calendar Quarter			0.990***

^*^Indicates significance at the 0.05 level

^***^Indicates significance at the 0.001 level

### Sensitivity analyses

Both sensitivity analyses showed similar results to the main analysis (70% population cutoff, *N* = 26) in terms of the directionality and significance of the relation between the various forms of non-lethal VAW and stay-at-home order duration (results available on request). Different from the main analysis, in the 60% cutoff sensitivity analysis (*N* = 28), the negative association between sexual IPV and duration of stay-at-home orders remained significant when adjusting for population size and seasonality (IRR, 0.999; 95% CI, 0.998, 0.999). Also different from the main analysis, in the 80% cutoff sensitivity analysis (*N* = 22), the negative association between psychological IPV and duration of stay-at-home orders was not significant (IRR 0.999; 95% CI, 0.999, 1.000).

## Discussion

### Summary and interpretation

This analysis is the most comprehensive assessment of the impacts of jurisdictional-level COVID-19 stay-at-home orders on lethal and non-lethal forms of VAW in the U.S. The analysis leverages a natural experimental design to compare pre-pandemic incidents of both forms of VAW during 2019 with those in 2020, the most intense period of implementation of COVID-19 risk-mitigation measures. As a result, the analysis was designed to capture the impacts of these measures at the height of the COVID-19 public health crisis. Findings offer insights about population-level shifts in VAW that may occur during periods of acute national and global crisis.

The results from the count models indicate that COVID-19 NPRM strategies, as measured in quarterly duration in days at the jurisdictional level, were not associated with quarterly counts of femicide after adjusted for seasonal effects and estimated jurisdictional population. Although there is evidence of increased rates of femicide in 2020 [31], this study is the first to show lower-than-projected rates of femicide associated with pandemic-related stay-at-home orders. The finding from the adjusted models of no increase in incidence of death by femicide with stay-at-home order duration contradicts evidence of increases during the pandemic in rates of non-lethal IPV [11], which often is a precursor to femicide. Our own analyses indicated an increase in incidence of reported physical IPV and economic IPV with stay-at-home orders. The increase in both physical IPV and economic IPV could have been due to pandemic-related shifts in the distribution of available economic resources between women and their partners [50] in addition to women becoming more vulnerable to assaults due to social isolation [26, 27]. Interestingly, we found both psychological and sexual IPV to be negatively associated with pandemic-related stay-at-home disorders, indicating perhaps a decrease in reporting during lockdowns. However, these findings need further study in light of evidence from other investigators showing a complex association between COVID-19 related distress and conflict and both psychological and sexual IPV victimization. For example, one study showed that higher levels of COVID-19 distress related to family relationships and higher levels of conflict about COVID-19 correlated positively with psychological IPV victimization, whereas higher levels of distress associated with friendships were correlated with a reduced likelihood of this form of IPV, and further no association was found between any type of COVID-19 related distress or conflict and sexual IPV victimization [51]. The combination measure of all forms of IPV was positively associated with the duration of stay-at-home orders, indicating a net increase in risk of IPV during lockdowns. Reported non-partner sexual violence was found to be negatively associated with duration of stay-at-home orders, which may indicate a decrease in reporting or even a decrease in exposure to non-intimate potential offenders during lockdowns. Whereas the sensitivity analyses showed differences for the sexual IPV and psychological IPV models, we are unable to attribute this variation to differences among jurisdictions or population sizes as both were changed in the analyses as a result of changing the inclusion criteria.

### Implications of the findings in context of existing literature

The findings of this analysis have important implications for future research and public health responses during periods of social and public health crisis. First, to the extent possible, this analysis should be expanded to understand the impacts of various combinations of jurisdictional NPRM strategies on incidents of lethal and on-lethal forms of VAW. Second, efforts are needed to understand whether and to what extent any concurrent shifts in investment in VAW prevention and response may have been effective. Third, research is needed to understand the potentially differential effects by race, ethnicity, income, and other markers of vulnerability of jurisdictional NPRM strategies on VAW [52]. Fourth, within specific jurisdictions, where stay-at-home orders and other NPRM strategies varied substantially across cities or counties, research is needed to understand the impacts of local risk-mitigation strategies on VAW, overall and again by markers of vulnerability. Fifth, the finding from this analysis of increased rates of non-lethal VAW at the height of COVID-19 NPRM efforts imply subsequent elevated rates of population-level disease conditions, such as poor mental health, poor sexual and reproductive health, cardiovascular conditions, and years of potential life lost [53], as the risks of these conditions are heightened with exposure to violence against women [54–56]. Importantly, the health impacts of elevated partner and non-partner violence during the COVID-19 pandemic are likely to be experienced disproportionately by women and likely by women of color specifically, possibly increasing gender and racial health disparities associated with exposure to IPV [53, 57]. Further analysis is warranted to understand the intersectional impacts of COVID-19 NPRM strategies during this period. Thus, the clear benefits of COVID-19 risk-mitigation strategies on averting disease and death due to COVID-19 should be weighed against their costs with respect to their collateral health impacts through increased exposure to IPV.

In general, our analytical strategy provides a useful methodological roadmap about how to assess quantitatively the effects of major public health crises on incidents of lethal and non-lethal forms of violence against women. A critical next step in this research agenda is to ensure the availability of timely and comprehensive data on incidence of exposure to these forms of violence to provide accurate estimates of effects.

### Study limitations

Some study limitations are notable and offer insights for future research. First, while the availability of mortality data allowed for the inclusion of 51 unique jurisdictions in the femicide analysis, the incomplete reporting by police precincts limited availability of jurisdiction-level data on non-lethal VAW to 26 jurisdictions. Even within these jurisdictions, only two had 100% reporting from the local police agencies. As a result, the findings presented here are not generalizable to the national level. Importantly, state jurisdictions that had some of the longest and expansive stay-at-home orders (i.e., California, New Jersey, New York, Pennsylvania) were not included in the analysis due to a failure to report crime data to the NIBRS. Second, the jurisdictions included in the main analysis of non-lethal forms of VAW were those that reported such incidents to the NIBRS in all 24 months during the 2019-2020 period of analysis and that had at least 70% of the jurisdiction’s population being included in the catchment area of the agencies reporting. The self-selection of reporting agencies may have biased the estimated impacts of stay-at-home orders for the jurisdictions represented in the analysis. To assess the magnitude of agency self-selection on the risk of this source of bias, sensitivity analyses suggested similar results to the main analysis in terms of the directionality and significance of the relationship between the various forms of non-lethal VAW and jurisdictional stay-at-home order duration. This finding was true except in the case of sexual IPV, which had a stronger association in the sensitivity analysis with more jurisdictions and in the case of psychological IPV which lost its significance in the more conservative sensitivity analysis. Third, the possible misclassification of lethal and non-lethal incidents of VAW as non-gendered forms of violence cannot be ruled out. This source of bias would tend to attenuate observed relationships of interest toward the null, such that the findings presented here would be lower bounds of the relationships of interest. Furthermore, the NIBRS provides data only on crimes reported to authorities. As such, many incidents of VAW may go unreported, particularly during a time of mandated social isolation. In analyses using self-report data from the National Crime Victimization Survey (NCVS) before the pandemic, half of all of intimate partner violence was estimated to go unreported [58]. However, the NCVS was not an appropriate data set to use for this study, as it is not representative at the state level [59, 60]. Fourth, the existence on the books of jurisdictional stay-at-home orders may not capture the intensity of their implementation across jurisdictions, nor corresponding investments by some jurisdictions to mitigate the impacts of stay-at-home orders on VAW. Fifth, on a similar note, the duration and intensity of other jurisdictional NPRM strategies, such as business and school closures, is not captured in this analysis. It is possible that the combination of jurisdictional orders may have varied differently across jurisdictions in this analysis, and the duration of these orders combined may have been related differently to the VAW outcomes measured here. Finally, the duration and intensity of stay-at-home orders and other NPRM strategies may have varied *within* jurisdictions in ways that were not captured in this jurisdiction-level analysis. Finally, the analysis of VAW as a function of jurisdiction-level-stay-at-home executive orders did not account for variability in the rates of each outcome by relevant sociodemographic characteristics such as race, education, or neighborhood context. The risks of femicide and non-lethal assault by an intimate partner vary by these characteristics [1, 16, 20, 31], and future research should consider intersectional differences in the risk of VAW, and factors that mediate the stay-at-home orders.

## Conclusion

In 26 jurisdictions of the U.S. during 2020, jurisdictional stay-at-home orders to curb the transmission of COVID-19 appear to have had adverse effects on non-lethal physical and economic forms of VAW and that the risk of non-partner forms of sexual VAW may have been reduced, possibly due to women’s reduced exposure to potential non-partner perpetrators during the most intense periods of lockdown. Nationally representative data on incidents of lethal and non-lethal VAW are needed to understand the full nature and scope of these effects for women and potentially heterogeneous effects among women in the US. Moreover, the long-term effects on women’s health of NPRM-related VAW need to be studied, given the unfolding nature of pandemic-related stressors like ongoing economic hardship and substance misuse. The immediate and long-term impacts on VAW of acute shocks, like pandemics, natural disasters, and humanitarian emergencies, is an important field of future investigation to ensure adequate protections are put in place to mitigate the collateral impacts of crisis response efforts on the incidence of violence against women.

### Supplementary Information

**Additional file 1:** **Appendix A. **Jurisdiction Inclusion Criteria for NIBRS analyses. **Appendix B. **Sensitivity Analyses.

## Data Availability

The datasets used during the current study are available publicly from the following repositories: • Murder Accountability Project: https://www.murderdata.org/p/data-docs.html • National Incident-Based Reporting System ICPSR Repository https://www.openicpsr.org/openicpsr/project/100707/version/V17/view • Ballotpedia https://ballotpedia.org/States_that_issued_lockdown_and_stay-at-home_orders_in_response_to_the_coronavirus_(COVID-19)_pandemic,_2020 The compiled dataset generated during analysis are available from the corresponding author on reasonable request.

## Abbreviations

CI: Confidence intervals
COVID-19: Coronavirus disease of 2019
IPV: Intimate partner violence
IRR: Incident rate ratios
MAP: Murder Accountability Project
NCVS: National Crime Victimization Survey
NIBRS: National Incident-Based Reporting System
NPRM: Non-pharmaceutical risk-mitigation
U.S.: United States of America
VAW: Violence Against Women

## Footnotes

1 Hotline calls are a complex way to understand the problem, because women experiencing lockdown with a perpetrator of violence may not feel safe to call and may have been cut off for some period from protective services.

2 Yearly jurisdictional-level female population estimates were based on projections from the 2010 census population for each jurisdiction. The median jurisdictional population was applied to each quarter of the year so there was a stable population size equivalent to the median for the year.
