# Supplementary material for: Collateral effects of COVID-19 stay-at-home orders on violence against women in the United States, January 2019 to December 2020
Source: BMC Public Health. 2024 Jan 2;24:51. doi: 10.1186/s12889-023-17546-y (PMC10763052; doi:10.1186/s12889-023-17546-y)
Supplement: Supplementary file 1 — Additional file 1: Appendix A. Jurisdiction Inclusion Criteria for NIBRS analyses. Appendix B. Sensitivity Analyses. [file 12889_2023_17546_MOESM1_ESM.docx]

| **Appendix A: Jurisdiction Inclusion Criteria for NIBRS analyses** | | |
| --- | --- | --- |
| **Jurisdiction Name** | **Reported for entire 24-month period** | **% of state population in catchment area*** |
| Alabama | Yes | - |
| Alaska | No | - |
| Arizona | Yes | 11.04% |
| Arkansas | Yes | 89.04% |
| California | No | - |
| Colorado | Yes | 93.73% |
| Connecticut | Yes | 93% |
| Delaware | Yes | 100% |
| District of Columbia | Yes | N/A± |
| Florida | No | - |
| Georgia | Yes | 21.10% |
| Hawaii | Yes | 69.74% |
| Idaho | Yes | 83.85% |
| Illinois | Yes | 1.88% |
| Indiana | Yes | 37.55% |
| Iowa | Yes | 87.25% |
| Kansas | Yes | 72.20% |
| Kentucky | Yes | 95.58% |
| Louisiana | Yes | 35.48% |
| Maine | Yes | 37.14% |
| Maryland | Yes | 17.11% |
| Massachusetts | Yes | 84.18% |
| Michigan | Yes | 88.17% |
| Minnesota | Yes | 80.35% |
| Mississippi | Yes | 26.55% |
| Missouri | Yes | 47.50% |
| Montana | Yes | 91.41% |
| Nebraska | Yes | 51.83% |
| Nevada | No | - |
| New Hampshire | Yes | 88.44% |
| New Jersey | No | - |
| New Mexico | Yes | 38.06% |
| New York | No | - |
| North Carolina | Yes | 78.59% |
| North Dakota | Yes | 98.82% |
| Ohio | Yes | 75.36% |
| Oklahoma | Yes | 53.53% |
| Oregon | Yes | 88.54% |
| Pennsylvania | No | - |
| Rhode Island | Yes | 98.23% |
| South Carolina | Yes | 97.30% |
| South Dakota | Yes | 77.53% |
| Tennessee | Yes | 99.19% |
| Texas | Yes | 63.79% |
| Utah | Yes | 88.85% |
| Vermont | Yes | 92.11% |
| Virginia | Yes | 98.40% |
| Washington | Yes | 83.30% |
| West Virginia | Yes | 47.97% |
| Wisconsin | Yes | 100% |
| Wyoming | Yes | 8.77% |
| *Population estimates based on 2010 Census estimates. Local agency population data obtained from [Law Enforcement Agency Identifiers Crosswalk Series](https://www.icpsr.umich.edu/web/ICPSR/series/366)  ± D.C. was excluded as the only reporting agency was the Metro Transit Police Department, the police agency of the Washington Metropolitan Area Transit Authority. It is unclear what percentage of the population is captured under this jurisdiction. | | |

**Appendix B – Sensitivity Analyses**

| **Table B1 – 60% cutoff**  Incident rate ratios with fixed effects for quarterly jurisdiction-specific counts of non-lethal VAW by quarterly duration in days of jurisdiction-level-stay-at-home executive orders, 2019-2020 (n=28) | | | |
| --- | --- | --- | --- |
|  | Unadjusted | Adjusted for population | Adjusted for population and seasonal effects |
| ***Physical IPV*** |  |  |  |
| Stay-at-home order duration | 1.001*** | 1.001*** | 1.001*** |
| Calendar Quarter |  |  | 1.016*** |
| ***Psychological IPV*** |  |  |  |
| Stay-at-home order duration | 0.999** | 0.999*** | 0.999*** |
| Calendar Quarter |  |  | 1.012*** |
| ***Economic IPV*** |  |  |  |
| Stay-at-home order duration | 1.003*** | 1.003*** | 1.001*** |
| Calendar Quarter |  |  | 1.076*** |
| ***Sexual IPV*** |  |  |  |
| Stay-at-home order duration | 0.999* | 0.999* | 0.999* |
| Calendar Quarter |  |  | 0.995* |
| ***Any IPV*** |  |  |  |
| Stay-at-home order duration | 1.001*** | 1.001*** | 1.001*** |
| Calendar Quarter |  |  | 1.021*** |
| ***Sexual Violence (Non-partner)*** |  |  |  |
| Stay-at-home order duration | 0.997*** | 0.997*** | 0.997*** |
| Calendar Quarter |  |  | 0.991*** |
| *Indicates significance at the 0.05 level. **Indicates significance at the 0.01 level. ***Indicates significance at the 0.001 level | | | |

| **Table B2 – 80% cutoff**  Incident rate ratios with fixed effects for quarterly jurisdiction-specific counts of non-lethal VAW by quarterly duration in days of jurisdiction-level-stay-at-home executive orders, 2019-2020 (n=22) | | | |
| --- | --- | --- | --- |
|  | Unadjusted | Adjusted for population | Adjusted for population and seasonal effects |
| ***Physical IPV*** |  |  |  |
| Stay-at-home order duration | 1.001*** | 1.001*** | 1.001*** |
| Calendar Quarter |  |  | 1.011*** |
| ***Psychological IPV*** |  |  |  |
| Stay-at-home order duration | 1.000 | 1.00 | 0.999 |
| Calendar Quarter |  |  | 1.020*** |
| ***Economic IPV*** |  |  |  |
| Stay-at-home order duration | 1.003*** | 1.003*** | 1.001*** |
| Calendar Quarter |  |  | 1.061*** |
| ***Sexual IPV*** |  |  |  |
| Stay-at-home order duration | 0.999* | 0.999* | 0.999 |
| Calendar Quarter |  |  | 0.989* |
| ***Any IPV*** |  |  |  |
| Stay-at-home order duration | 1.001*** | 1.001*** | 1.001*** |
| Calendar Quarter |  |  | 1.017*** |
| ***Sexual Violence (Non-partner)*** |  |  |  |
| Stay-at-home order duration | 0.997*** | 0.997*** | 0.997*** |
| Calendar Quarter |  |  | 0.990*** |
| *Indicates significance at the 0.05 level. **Indicates significance at the 0.01 level. ***Indicates significance at the 0.001 level | | | |
